# Insights into Metabolic Activity and Structure of the Retina through Multiphoton Fluorescence Lifetime Imaging Microscopy in Mice

**DOI:** 10.3390/cells11152265

**Published:** 2022-07-22

**Authors:** Niranjana Kesavamoorthy, Jason A. Junge, Scott E. Fraser, Hossein Ameri

**Affiliations:** 1 Department of Ophthalmology, USC Roski Eye Institute, Keck School of Medicine, University of Southern California, Los Angeles, CA 90033, USA; niranjana.kesavamoorthy@med.usc.edu; 2 Department of Biological Sciences, David Dornsife College of Letters Arts and Sciences, University of Southern California Dana, Los Angeles, CA 90089, USA; junge@usc.edu (J.A.J.); sfraser@provost.usc.edu (S.E.F.)

**Keywords:** retina, multiphoton fluorescence lifetime imaging microscopy, FLIM, NAD(P)H, metabolic imaging, glycolysis, oxidative phosphorylation

## Abstract

Fluorescence lifetime imaging microscopy (FLIM) evaluates the metabolic state of tissue based on reduced nicotinamide adenine dinucleotide (NAD(P)H) and flavin adenine dinucleotide (FAD). Fluorescence lifetime imaging ophthalmoscopy (FLIO) can image the fundus of the eyes, but cannot detect NAD(P)H. We used multiphoton FLIM to study the metabolic state of the retina in fixed eyes of wild-type mice C57BL6/J. We sectioned the eye using a polyacrylamide gel-embedding technique and estimated the percentage of bound NAD(P)H. We found that oxidative phosphorylation was the predominant metabolic state, particularly in the inner retina, when a fixed retina was used. We also demonstrated the feasibility of FAD imaging of the retina. In addition, we demonstrated that autofluorescence and various FLIM channels, such as hemoglobin, melanin and collagen, can be used to evaluate the structure of the retina and other parts of the eye without any special staining.

## 1. Introduction

Fluorescence lifetime imaging microscopy (FLIM) displays the fluorescence lifetimes of various fluorophores in a sample as a two-dimensional image. Unlike intensity-based images, fluorescence lifetimes depend not on the fluorophore concentration but on the local environment. The same fluorophore could have different lifetimes based on its interacting molecule [1]. Using FLIM, one can estimate the metabolic state of the tissue based on the fluorescence lifetimes of the free and bound reduced nicotinamide adenine dinucleotide (NAD(P)H) [1,2]. The lifetime of free NAD(P)H is smaller than the protein-bound NAD(P)H [1]. The presence of free NAD(P)H in tissue suggests glycolysis, whereas the presence of bound NAD(P)H signals oxidative phosphorylation [3]. Previous studies have used FLIM to determine a predominance of glycolysis or oxidative phosphorylation by measuring the amounts of free and bound NAD(P)H in various tissues, including human organoid [2,4,5]. FLIM can be performed using a multiphoton or confocal approach [6]. The advantages of the multiphoton approach over the confocal method are that the light penetrates deeper and photodamage is limited because only the required sample volume is excited, preventing light scatter and absorption before reaching the region of interest [7,8,9].

FLIM has been used to study eyes. Some studies have used fluorescence lifetime imaging ophthalmoscopy (FLIO), a combination of FLIM and confocal laser scanning ophthalmoscopy, to image eyes in vivo; however, NAD(P)H cannot be imaged by FLIO [10,11,12,13]. Previous investigators have imaged ex vivo, unfixed porcine eyes, using two-photon FLIM to investigate the fundus and to assess the morphology and metabolism of the cornea and lens [14,15]. In this article, we demonstrate that FLIM can be used to study the metabolic status of the retina as well as the structure of the retina and other parts of the eye.

## 2. Materials and Methods

### 2.1. Animals

All procedures complied with Institutional Animal Care and Use Committee (IACUC) guidelines at the University of Southern California. Wild-type mice (C57BL6/J) were purchased from Jackson Laboratories (Bar Harbor, ME, USA). Mice were housed in a 12-h light/dark cycle and food and water were provided ad libitum.

### 2.2. Enucleation and Fixation of Eyes

Animals were euthanized, and the eyeballs were enucleated. Eleven eyes were included in the study. Before enucleation, the superior portion of the cornea was marked with cautery to preserve the orientation of the eyeballs. The eyeballs were punctured and placed in 4% paraformaldehyde for one hour. The corneas were then removed and the eyecups were placed in 4% paraformaldehyde for two hours.

### 2.3. Embedding and Sectioning

The eyecups were embedded in polyacrylamide gel, based on Hayaran et al.’s method [16]. The gel was prepared by mixing 3 mL 40% Bisacrylamide, 6 mL ultrapure Milli-Q water, 1 mL 10× Tris Buffered Saline, and 47 μL 10% Ammonium Persulfate. The eyecups were placed in this gel mixture, their lens removed within the gel mixture, and 30 μL Tetramethylethylenediamine was then added (Supplementary Figure S1). Sectioning of the embedded eyecups was performed using Leica Microtome VT1200 (Leica Microsystems, Deerfield, MA, USA). Sections between 100 μm and 200 μm thick were made. In half of the samples, the eyes were oriented to obtain superior–inferior sections; in the other half, nasal-temporal sections were obtained. In each eye, the largest central sections that included the optic nerve and peripheral retina were selected for imaging.

### 2.4. Multiphoton FLIM Imaging

The Leica SP8 DIVE FALCON (Leica Microsystems, Deerfield, MA, USA) was used for imaging; the samples were excited at 740 nm for metabolic and intrinsic autofluorescence signal imaging and 860 nm for second harmonic generation (SHG) at ~500 μW laser power for each wavelength. Autofluorescence was collected on three channels of hybrid detectors: 425–475 nm for NAD(P)H, 502–577 nm for flavin adenine dinucleotide (FAD), and 600–650 nm for hemoglobin and melanin. The NAD(P)H signal was excited through non-linear, multiphoton excitation by a Spectra Physics InSight X3 tunable IR laser (MKS Spectra-Physics, Milpitas, CA, USA) and emission was collected from 425–475 nm through adjustment of the Leica 4Tune NDD filtering system (Leica Microsystems, Deerfield, MA, USA). Light of wavelengths 475+ nm was excluded from detection, because other autofluorescent molecules, such as FAD, are multiphoton excitable at 740 nm and we wanted to exclude those signals in our analysis of NAD(P)H. The SHG of collagen was collected from 411 to 463 nm. The choice of excitation and emission spectra were based on our previous work and the work of others [4,17,18,19]. We used a Leica 40×/1.2 NA CSII water immersion objective (Leica Microsystems, Deerfield, MA, USA), and a single focal plane image was chosen ten microns deep in the retina sections for standardization. For three-dimensional retina images to detect vascular networks, we collected z-stacks from the entire thickness of the section with optical sectioning at Nyquist, and displayed in Imaris 64 bit 9.7.0 software (Bitplane, Belfast, UK).

### 2.5. FLIM Analysis

The vertical sections were divided into a superior and an inferior region, and the horizontal sections were split into temporal and nasal regions. Each region was classified into five zones: far periphery (near the ora serrata), mid periphery (halfway between the ora serrata and the optic disc), peripapillary (200 μm from the optic disc), central (400 μm from the optic disc), and paracentral (600 μm from the optic disc). Each zone was divided into outer (photoreceptor inner (IS), outer segments (OS), and outer nuclear layer (ONL)), inner (outer plexiform layer (OPL)), inner nuclear layer (INL), inner plexiform layer (IPL), ganglion cell layer (GCL), and retinal nerve fiber layer (RNFL)). The phasor approach to FLIM analysis [20,21] to determine percentages of bound NAD(P)H, was accomplished in Leica’s LASX FLIM FCS software. In this software package, a metabolic trajectory was drawn from one edge of the unit circle, from 0.4 ns lifetime (lifetime constant, τ, of 100% free NAD(P)H), passing through the center of mass, and extrapolated to an intersection with the edge of the unit circle (representing 100% bound NAD(P)H), using the ratiometric analysis tool. Next, regions of interest (ROI) were created in the retinal images to determine phasor positions for the pixels within the ROIs; we standardized ROIs to have a width of 150 μm. We then moved the ratiometic slider along the metabolic trajectory we drew in the phasor, over the center of mass for the phasor distribution for each ROI. (Supplementary Figure S2). The bound NAD(P)H %, which is the ratio of bound NAD(P)H / total NAD(P)H multiplied by 100, is calculated from the position of each ROI’s center of mass along the metabolic trajectory. The phasor position for melanin was determined based on a previous study [22]. SHG Leica’s DIVE FALCON is designed as a spectral + lifetime imaging instrument and can therefore separate collagen autofluorescence from second harmonic signal. The second harmonic signal of collagen has an extremely short lifetime, approaching instantaneous, and therefore, a distinct phasor position which is far from collagen autofluorescence [18]. Thus, we can infer that the signal we obtained from collagen is due to second harmonic and not due to autofluorescence.

To determine the phasor position for hemoglobin, we selected an ROI in images corresponding to a retinal blood vessel, and by using the phasor ROI selection tool, we then highlighted all pixels within a retinal image containing that specific phasor lifetime position for hemoglobin.

### 2.6. Statistical Analysis

The mean, standard deviation (SD), and SEM for the percentage of bound NAD(P)H were obtained. Two-tailed paired *t*-tests were used to compare the percentage of bound NAD(P)H. 

## 3. Results

### 3.1. Metabolic Imaging

#### 3.1.1. NAD(P)H Channel

**Figure 1:**
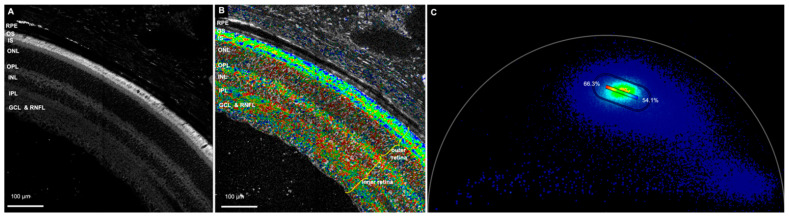
Retina in the NAD(P)H channel. (**A**) Autofluorescence image of the retina. (**B**) The NAD(P)H channel. The blue spots indicate areas with 54.1% bound NAD(P)H, and the red spots show areas with 66.3% bound NAD(P)H. As is evident, the outer retina is more towards the blue spectrum, and the inner retina is more towards the red spectrum, indicating less bound NAD(P)H in the outer retina. (**C**) Phasor plot for the above image showing rainbow spectrum from red to blue. (RPE—retinal pigment epithelium, OS—photoreceptor outer segment, IS—photoreceptor inner segment, ONL—outer nuclear layer, OPL—outer plexiform layer, INL—inner nuclear layer, IPL—inner plexiform layer, GCL—ganglion cell layer, RNFL—retinal nerve fiber layer, NAD(P)H —reduced nicotinamide adenine dinucleotide).

**Figure 2:**
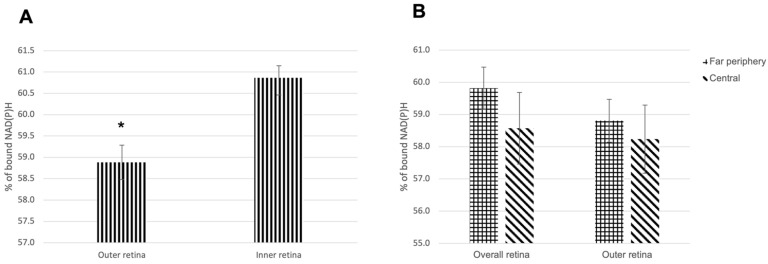
(**A**) Percentage of bound NAD(P)H in the outer retina vs. the inner retina. (**B**) Percentage of bound NAD(P)H in the far periphery compared to the central zones in the overall and the outer retina. Data are presented as mean ± SEM. (NAD(P)H-reduced nicotinamide adenine dinucleotide). * *p* < 0.0001.

In the NAD(P)H channel (425–475 nm emission spectra), we compared the percentage of bound NAD(P)H between the outer retina (photoreceptor inner and outer segments and outer nuclear layer) and the inner retina (outer plexiform layer, inner nuclear layer, inner plexiform layer, ganglion cell layer, and retinal nerve fiber layer) (Figure 1 and Table S1). The zones with artifacts, damages and folds were excluded. The mean ± SDs of the bound NAD(P)H percentage in the outer and inner retina were 58.9 ± 3.7 and 60.9 ± 2.6, respectively. The total number of images used was 86. The total number of eyes imaged in the NAD(P)H channel was 11 (Figure 2A). Statistical analysis revealed a highly statistically significant difference in the percentage of bound NAD(P)H between the outer and inner retina (*p* < 0.0001).

Next, we compared the percentage of bound NAD(P)H in the far periphery with the central zones to determine whether the density of photoreceptors and ganglion cells has any effects on metabolic activity (Table S2 and Supplementary Figure S3). The comparison was made in two different ways: the percentage of bound NAD(P)H in the overall retina of the two zones and the percentage of bound NAD(P)H in the outer retina of the two zones. In the overall retina, the mean ± SD bound NAD(P)H percentage was 59.8 ± 2.5 in the far periphery and 58.6 ± 4.3 in the central zones (*p* = 0.36). In the outer retina, the mean ± SD bound NAD(P)H percentage was 58.8 ± 2.6 in the far periphery and 58.2 ± 4.1 in the central zones (*p* = 0.64) (n = 15) (Figure 2B). Neither comparisons demonstrated a statistically significant difference between the far periphery and central retina.

#### 3.1.2. FAD Channel

**Figure 3:**
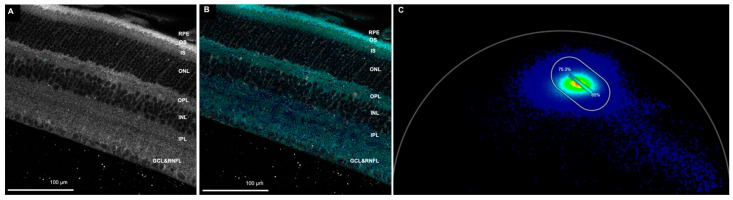
Retina in the FAD channel. (**A**) Autofluorescence image of the retina. (**B**) The FAD channel. The dark blue indicates less bound FAD than cyan. The dark blue spots indicate areas with 75.3% free FAD, and the cyan spots show areas with 65% free FAD. (**C**) Phasor plot showing spectrum from dark blue to cyan. A metabolic trajectory was drawn from one edge of the unit circle from 2.6 ns lifetime (lifetime constant, τ, of 100% free FAD), passing through the center of mass, and extrapolated to an intersection with the edge of the unit circle (representing 100% bound FAD), using the ratiometric analysis tool. The color bar was marked on top of the trajectory to indicate the free and bound FAD. (RPE-retinal pigment epithelium, OS—photoreceptor outer segment, IS—photoreceptor inner segment, ONL—outer nuclear layer, OPL—outer plexiform layer, INL—inner nuclear layer, IPL—inner plexiform layer, GCL—ganglion cell layer, RNFL—retinal nerve fiber layer, FAD—flavin adenine dinucleotide). Scale bar: 100 µm.

We were also able to image FAD, which could also be used to study metabolic state. For the FAD imaging, we used 502–577-nm emission spectra (Figure 3).

### 3.2. Structural Imaging

**Figure 4:**
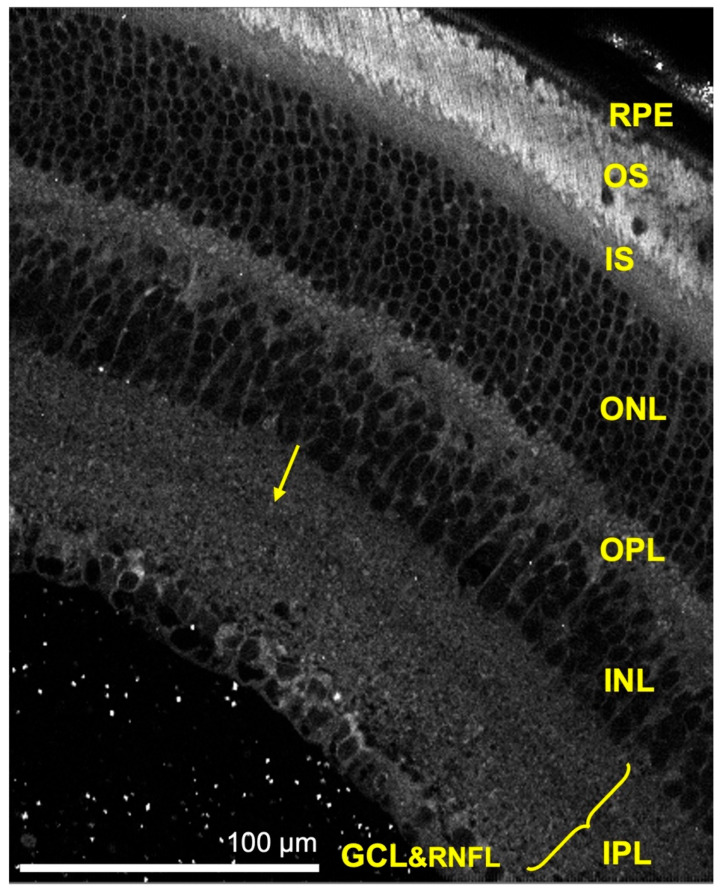
Autofluorescence image of the retina showing the retinal layers. The yellow arrow indicates the hypo-autofluorescent band in the inner plexiform layer. (RPE—retinal pigment epithelium, OS—photoreceptor outer segment, IS—photoreceptor inner segment, ONL—outer nuclear layer, OPL—outer plexiform layer, INL—inner nuclear layer, IPL—inner plexiform layer, GCL—ganglion cell layer and RNFL—retinal nerve fiber layer). Scale bar: 100 μm.

Autofluorescence images could determine the retinal structure by delineating the retinal layers. All the retina layers could be visualized, except the internal limiting membrane and the external limiting membrane. We also visualized a distinct band of relative hypo-autofluorescence in the inner plexiform layer, which is not visible on bright-field microscopy or common structural retinal staining, such as Haematoxylin and Eosin, and Periodic Acid Schiff (Figure 4). Using multiphoton FLIM, we were able to evaluate other fluorophores, such as melanin, hemoglobin and collagen.

#### 3.2.1. Hemoglobin and Melanin Channel

**Figure 5:**
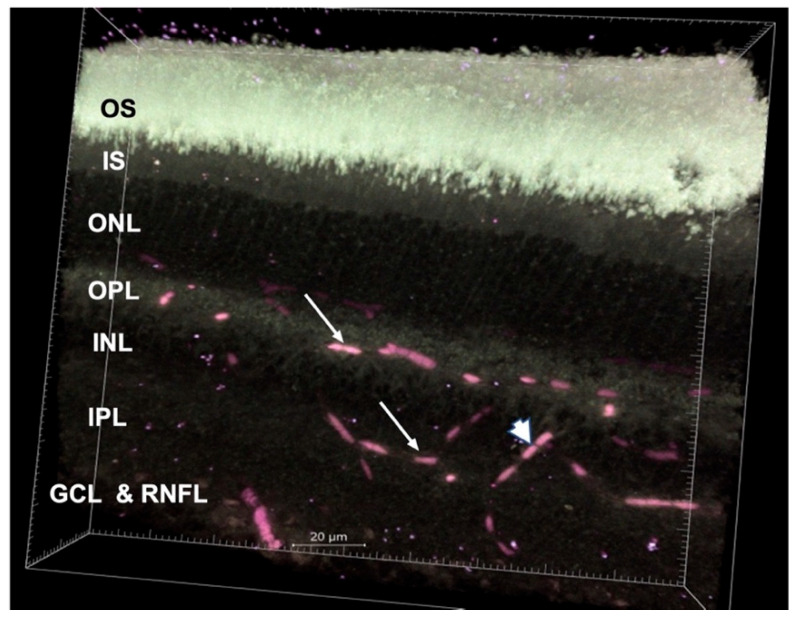
3D image of the retina obtained by z-stacking (thickness—200 μm). The arrows indicate the deep and superficial capillary plexus, and the arrowhead shows a penetrating vessel connecting the two plexuses. The image reveals all the retinal layers except RPE (RPE was clipped out). (RPE—retinal pigment epithelium, OS—photoreceptor outer segment, IS—photoreceptor inner segment, ONL—outer nuclear layer, OPL—outer plexiform layer, INL—inner nuclear layer, IPL—inner plexiform layer, GCL-ganglion cell layer, RNFL-retinal nerve fiber layer). Imaris software was used to generate the 3D image.

**Figure 6:**
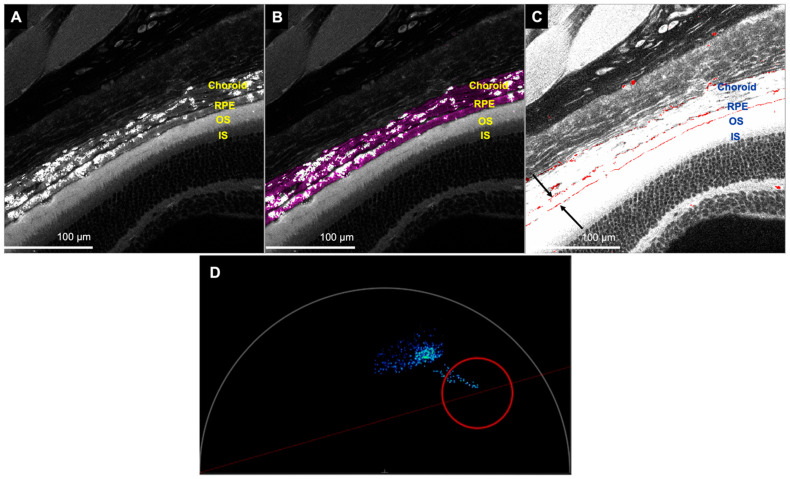
Representative images of the retina and choroid showing melanin and hemoglobin signals. (**A**) Autofluorescence image to show outline of the RPE and photoreceptors and determine the exact location of the melanin and hemoglobin signals. (**B**) Image displaying the melanin signal in the choroid and the RPE (shown in pink). (**C**) Image showing the hemoglobin signal in basal portion of the RPE (top arrow), apical portion of the RPE (bottom arrow) and the choroid (shown in red). To better visualize the hemoglobin signal, the brightness of the image was increased. The above three images were obtained from the most superficial z-section, since the choroid and RPE appear darker in the deeper z-sections because of blockage. (**D**) Phasor position for hemoglobin signal (red circle). The blue stain corresponds to the region of interest selected. (RPE—retinal pigment epithelium, OS—photoreceptor outer segment, IS—photoreceptor inner segment).

We could detect hemoglobin signals within the retinal and choroidal vessels when imaged with 600–650 nm emission spectra. A 3D image of the retina with several Z-sections demonstrated a deep capillary plexus in the outer plexiform layer, a superficial capillary plexus in the junction of the inner nuclear layer and inner plexiform layer, and a superficial layer composed of retinal vessels in the ganglion cell layer (Figure 5). In addition, we found two distinct bands of hemoglobin signal in the basal and apical portions of the retinal pigment epithelium (RPE) (Figure 6C). 

**Figure 7:**
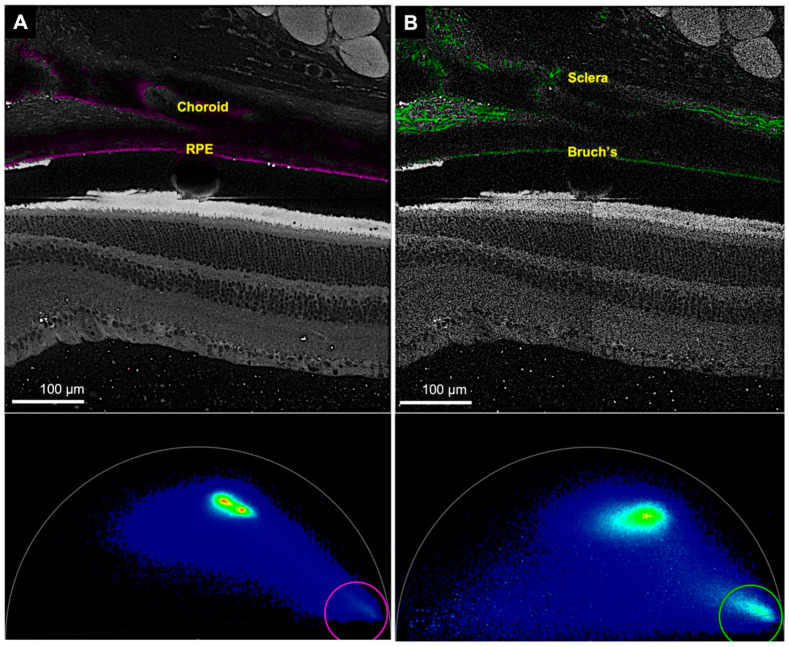
Representative images of the retina, choroid and sclera in the melanin and collagen channels. The same region was imaged in two different channels. (**A**) Top. Image showing the signal for melanin in the RPE and the choroid (in pink). Bottom. Phasor plot showing the phasor position of melanin (pink circle). (**B**) Top. The sclera and the Bruch’s membrane generate a second harmonic corresponding to collagen (in green). Bottom. Phasor representation of collagen (green circle). (RPE—retinal pigment epithelium). The gap between the RPE and photoreceptors is an artifact separation.

FLIM could also be used to study melanin. The RPE and the choroid displayed the melanin signal with the same emission spectra (600–650 nm) (Figure 6B and Figure 7A). 

#### 3.2.2. Collagen Channel

**Figure 8:**
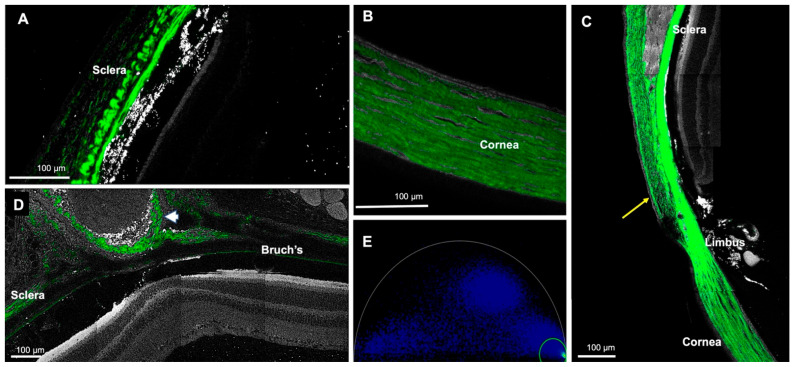
Representative collagen images from different regions generated by the second harmonic. (**A**) The sclera. Note the irregular arrangement and wavy fibers. (**B**) The cornea. The fibers are uniformly arranged. (**C**) The conjunctiva, sclera and cornea combined. The conjunctiva is indicated with a yellow arrow. The thickness of the sclera is greatest at the perilimbal region and decreases posteriorly. (**D**) The optic nerve sheath (indicated by a white arrowhead). (**E**) Representative phasor image for collagen (signal demonstrated in green).

The collagen channel could display the sclera, cornea, conjunctiva, episcleral tissue, optic nerve sheath, Bruch’s membrane, and iris stroma, all of which generate a second harmonic (Figure 7B and Figure 8) due to the fibrillar collagen [23]. The scleral fibers had an uneven thickness and irregular fiber arrangement, with the greatest thickness in the perilimbal region, which gradually diminished towards the posterior pole (Figure 8A,C). In contrast, the cornea had a uniform thickness, and the fibers had a regular structure (Figure 8B).

## 4. Discussion

In this article, we demonstrated that fixed mice eyes could be used for metabolic studies of the retina by measuring the percentages of free and bound NAD(P)H. We observed higher percentages of bound NAD(P)H in both the outer and inner retina, indicating a predominance of oxidative phosphorylation throughout the retinal thickness. Comparing the inner and outer retina, we noted a greater number of free NAD(P)H in the outer retina, indicating a higher level of glycolysis in the outer retina relative to the inner retina. The bound NAD(P)H percentages obtained in our study are specific to fixed retina. Since a previous study demonstrated that fixation increases fluorescence lifetimes, further studies are required to evaluate any differences between fresh and fixed retina [24]. Similar to our study, the article by Rueda et al. showed more glycolysis in the outer retina in mice (except in the photoreceptor outer segment). They also found an overall high oxidative phosphorylation (except in the outer photoreceptor segments and the inner nuclear soma), but the percentages of glycolysis and oxidative phosphorylation were not estimated [25]. Although fixation increases the fluorescence lifetimes, our findings are partially in agreement with a study by Browne et. al on human retinal organoids, which indicated a predominance of oxidative phosphorylation in the inner regions, but a predominance of glycolysis in the outer regions of the organoids [4]. Future studies on the human retina will determine whether its metabolic state is more similar to human retinal organoids, or to that found in mice. A study by Volland et al. found that in mice retina, the photoreceptor density was higher in the central regions compared to the periphery, with the highest photoreceptor density located 400 μm temporal to the optic disc; this region was defined as the area centralis [26]. Another study revealed that ganglion cell density was higher in the central retina than in the periphery [27]. To assess the potential correlation between the metabolic states of the retina and the cell density of photoreceptors and ganglion cells, we compared the metabolic states of the far periphery (near ora serrata), which has the lowest cell density, and the central zone (400 μm from the optic disc). These zones demonstrated no significant difference between them. 

We showed that a metabolic image of the retina could also be obtained with the FAD channel. However, in our study, we did not determine the fraction of bound and free FAD for our samples, since our focus was to determine the metabolic state of the retina using NAD(P)H. Previous studies have used FAD to study the metabolic state of cancer cells. Imaging with the FAD channel and the NAD(P)H channel can potentially be used to determine the ratio of bound NAD(P)H to bound FAD, which estimates the redox state of the tissue [2,28,29].

Using multiphoton FLIM, in addition to metabolic imaging, we performed structural imaging of the retina. We also showed that FLIM can be used for structural imaging of the sclera, cornea, choroid, optic nerve sheath, and the tissues surrounding the eyes, without any staining. FLIO can also be used to study the structure of the retina; however, unlike FLIM it cannot detect NAD(P)H in the fundus. This is due to the short emission spectra and excitation wavelength of NAD(P)H, and these short wavelengths are obstructed by the cornea and lens [10,11]. The autofluorescence images of the retina helped us to delineate the retinal layers. Interestingly, we noted a relative band of hypo-autofluorescence within the inner plexiform layer. Comparing our images to published immunochemical studies, which demonstrated specific immunostaining in the same region as our hypo-autofluorescent band, we speculate that the band corresponds to dendrites of type 2 catecholaminergic amacrine cells, neuronal junctions of some amacrine cells, ganglion cells, and bipolar cells [30,31,32]. Further histological studies are required to confirm this, which is beyond the scope of this study.

In FLIM, the hemoglobin channel can display retinal blood vessels well, particularly when a 3D video is generated from a thick retinal section. However, the vessels appear to be interrupted; this is due to the segmentation of the blood in the retinal vessels and capillaries, which occurs in tissues postmortem [33]. Similar to previous studies, we observed three vascular networks in the retina, a deep capillary plexus in the outer plexiform layer, a superficial capillary plexus in the junction of the inner nuclear layer and inner plexiform layer, and a superficial layer composed of retinal vessels in the ganglion cell layer [34,35]. Zimmerman et al. also demonstrated the same, but classified them into two layers [36]. We were also able to detect penetrating vessels between the layers. In addition to the retinal blood vessels, we found the presence of hemoglobin signal in the choriocapillaris. Although not performed in this study, intracardiac dye before euthanasia may allow for a better demonstration of vessels. Hemoglobin could also be present outside the blood vessels and, using FLIM, we detected its presence in the RPE. Tezel et al. have previously reported the presence of hemoglobin in the basal portion of the RPE and speculated that it could act as an oxygen reservoir during periods of increased oxygen demand [37]. Interestingly, in our study, in addition to a band of hemoglobin in the basal portion of the RPE, we noted an additional band in the apical portion.

FLIM also allows for the study of melanin distribution. In our study, a band of melanin signal corresponded to the RPE. Melanin signal was also detected in the choroid. Imaging melanin can be used to study diseases that affect the RPE, such as age-related macular degeneration and inherited retinal diseases.

This study demonstrated the additional possibility of imaging collagen by generating a second harmonic. We most likely detected type I collagen, since fibrillar collagen generates a second harmonic, and type I produces a prominent second harmonic [23,38]. In addition, type I collagen is the primary collagen in the cornea and sclera [39,40]. Thus, FLIM can illustrate the collagen architecture of the cornea and the sclera without any special stains. Collagen was detected in the choroid, including Bruch’s membrane, optic nerve sheath, episcleral tissue, conjunctiva, cornea, and iris stroma, but it was most prominent in the sclera. In line with these findings, previous studies have also imaged collagen in the sclera by generating a second harmonic in human and rabbit eyes [41,42].

A limitation of this study is that the imaging was performed on fixed tissues. We used fixed eyes for two reasons: (1) the soft consistency of the unfixed retina made it cumbersome to section, and (2) imaging the fresh retina resulted in artifacts; the fresh retinal sections disintegrated and the retinal architecture became distorted. We fixed the eyes by immersing in 4% paraformaldehyde for a total of 3 h.

In summary, our study determined the metabolic states and structure of fixed eyes from wild-type mice. We found that oxidative phosphorylation is the predominant metabolic state in the retina, but the outer retina shows significantly less oxidative phosphorylation than the inner retina. In addition, we were able to better localize hemoglobin that is present in the RPE. Multiphoton FLIM allowed for the study of the whole eye using NAD(P)H, FAD, hemoglobin, melanin, and collagen channels. In the future, with additional strategies, metabolic states of various disease models in mice can be determined using this study as a baseline.

## Data Availability

The data presented in this study are available in the supplementary materials and within the article. The supplementary data are also available in zenodo: https://doi.org/10.5281/zenodo.6820505.

## Supplementary Materials

The following supplementary materials can be downloaded at https://www.mdpi.com/article/10.3390/cells11152265/s1. Table S1: Percentage of bound NAD(P)H in the outer retina and inner retina; Table S2: Percentage of bound NAD(P)H in the overall retina and the outer retina (far periphery and central zones). Figure S1: Image showing the central section of the eyeball through the dissecting microscope; Figure S2: Illustration of phasor analysis; Figure S3: Intensity images of the far periphery and central zones; Figure S4: A2 distribution images of far periphery and central zones.
