# Supplementary material for: Insights into Metabolic Activity and Structure of the Retina through Multiphoton Fluorescence Lifetime Imaging Microscopy in Mice
Source: Cells. 2022 Jul 22;11(15):2265. doi: 10.3390/cells11152265 (PMC9331481; doi:10.3390/cells11152265)
Supplement: Supplementary file 1 [file cells-11-02265-s001.zip › cells-1799894-supplementary.pdf]

Supplementary Materials

Table S1. Percentage of bound NAD(P)H in the outer retina and inner retina

Location	Eye	Outer retina	Inner retina
Far periphery-superior	309-OD	55.1	59.2
	321-OD	56.1	58.1
	321-OS	63.3	63.3
	330-OD	60.4	61.7
	330-OS	62	64.45
Far periphery-inferior	309-OD	55.4	58.5
	321-OD	50.8	56.6
	321-OS	63.9	63.5
	330-OD	57.3	61.3
	330-OS	59.1	62.8
Mid periphery-superior	309-OD	55	59.25
	321-OD	60.3	63.6
	321-OS	59.3	62.75
	330-OD	59.5	60.6
	330-OS	60.9	61.7
Mid periphery-inferior	309-OD	56.4	60.5
	321-OD	62.4	60.9
	321-OS	62.8	63.25
	322-OD	57.7	59.2
	330-OD	61.1	60.2
	330-OS	61.6	61.15
Far periphery-temporal	324-OS	62.5	63.9
	414-OD	60.1	59.85
	414-OS	60.3	60.35
	418-OS	59.9	59.3
Far periphery-nasal	324-OD	58	59.95
	324-OS	60.8	63.6
	414-OD	57.8	59.85
	414-OS	59.8	60.2
	418-OS	53.6	56.55
Mid periphery-temporal	324-OS	61.5	63.15
	414-OD	61.7	58.6
	418-OS	58.5	58.15
Mid periphery-nasal	324-OD	56.2	58.55
	324-OS	61.6	63.45
	414-OD	62.3	59.35
	414-OS	62	62.35
	418-OS	56.1	57
Superior-peripapillary	309-OD	55.70	55.7
	330-OD	52.90	57.6
	330-OS	56.7	58.95
Superior-paracentral	309-OD	56.3	56.3
	330-OD	58.5	58.5
	330-OS	64	58.45
Inferior-peripapillary	309-OD	52.4	56.6
	330-OD	54.8	58.5
	330-OS	58	57.95

Inferior-paracentral	309-OD	54.3	57.6
	330-OD	57.9	63
	330-OS	57.9	58.55
Temporal-peripapillary	324-OD	57.5	60.6
	324-OS	61.4	62.05
	414-OD	62.3	64.75
	418-OS	62	64.05
	414-OS	52.4	62
Temporal-paracentral	324-OD	59.9	61.2
	324-OS	61.7	62.55
	414-OD	62.5	65.55
	418-OS	68.3	65.7
	414-OS	50.8	61.1
Nasal-peripapillary	324-OD	60.6	62.5
	324-OS	59.6	62.45
	414-OD	63.6	61.8
	418-OS	61.1	64.95
	414-OS	53.7	62.9
Nasal-paracentral	324-OD	61.9	60.35
	324-OS	59.8	63.4
	414-OD	65.4	63.3
	418-OS	62.2	65.55
	414-OS	50.8	64.25
Superior-central	309-OD	57.60	55.2
	330-OD	57.20	57.9
	330-OS	57.30	57.75
Inferior-central	309-OD	54.50	56.25
	330-OD	56.40	57.4
	330-OS	59.30	58
Temporal-central	324-OD	60.40	62.3
	324-OS	63.00	62.1
	414-OD	61.90	63.25
	418-OS	56.50	63.8
	414-OS	52.30	60.5
Nasal-central	324-OD	61.90	60.15
	324-OS	61.80	63.6
	414-OD	64.80	63.65
	418-OS	59.20	64.2
	414-OS	49.80	62.9

Table S2. Percentage of bound NAD(P)H in the overall retina and the outer retina (far periphery and central zones)

Location	Eye	Overall retina	Outer retina
Far periphery-superior	309-OD	58.2	55.1
	330-OD	61.8	60.4
	330-OS	62.2	62
Far periphery-inferior	309-OD	57.4	55.4
	330-OD	59.3	57.3
	330-OS	60.9	59.1
Far periphery-temporal	324-OS	63.5	62.5
	414-OD	59.5	60.1
	414-OS	60.6	60.3
	418-OS	59.9	59.9
Far periphery-nasal	324-OD	59.1	58
	324-OS	63.3	60.8
	414-OD	58	57.8
	414-OS	60.1	59.8
	418-OS	53.5	53.6
Superior-central	309-OD	57.60	57.60
	330-OD	57.20	57.20
	330-OS	58.00	57.30
Inferior-central	309-OD	54.50	54.50
	330-OD	57.10	56.40
	330-OS	59.30	59.30
Temporal-central	324-OS	62.20	63.00
	414-OD	61.90	61.90
	414-OS	52.30	52.30
	418-OS	56.00	56.50
Nasal-central	324-OD	61.30	61.90
	324-OS	63.30	61.80
	414-OD	64.80	64.80
	414-OS	49.80	49.80
	418-OS	63.30	59.20

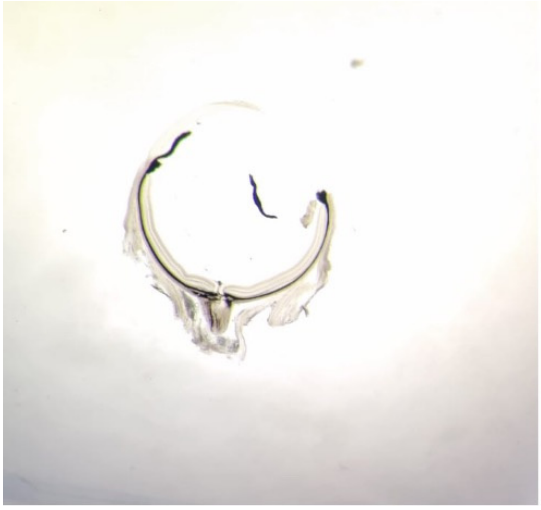

Figure S1. Image showing the central section of the eyeball through a dissecting microscope

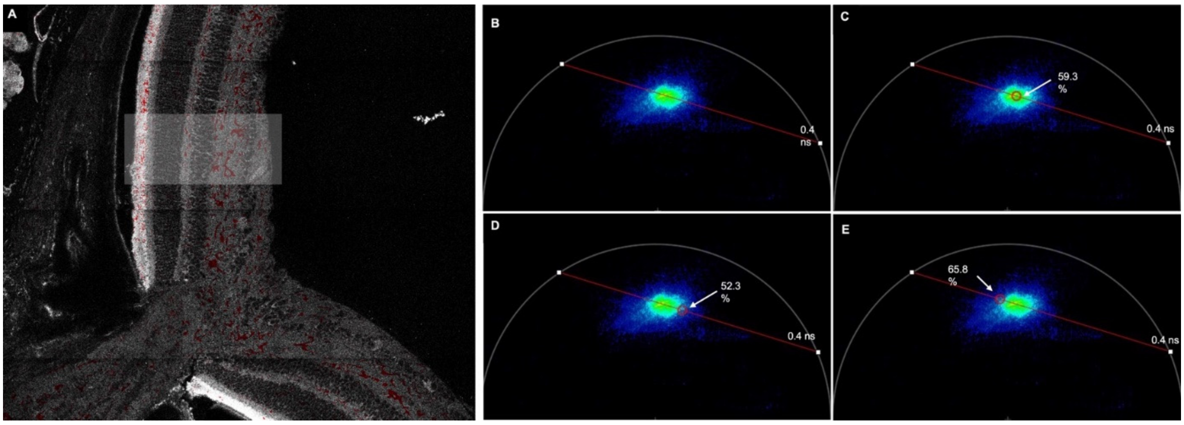

Figure S2. Illustration of phasor analysis (**A**)Image showing the region of interest selected for phasor analysis (shaded rectangle)

B-E) Phasor plot corresponding to the region of interest (**B**) a metabolic trajectory (red line) was drawn from one edge of the unit circle from 0.4 ns lifetime (lifetime constant, τ, of 100% free NAD(P)H), passing through the center of mass, and extrapolated to an intersection with the edge of the unit circle (representing 100% bound NAD(P)H) using the ratiometric analysis tool. (**C**)(**D**)(**E**) Phasor plots showing the percentage of bound NAD(P)H corresponding to the selected region (red circle).

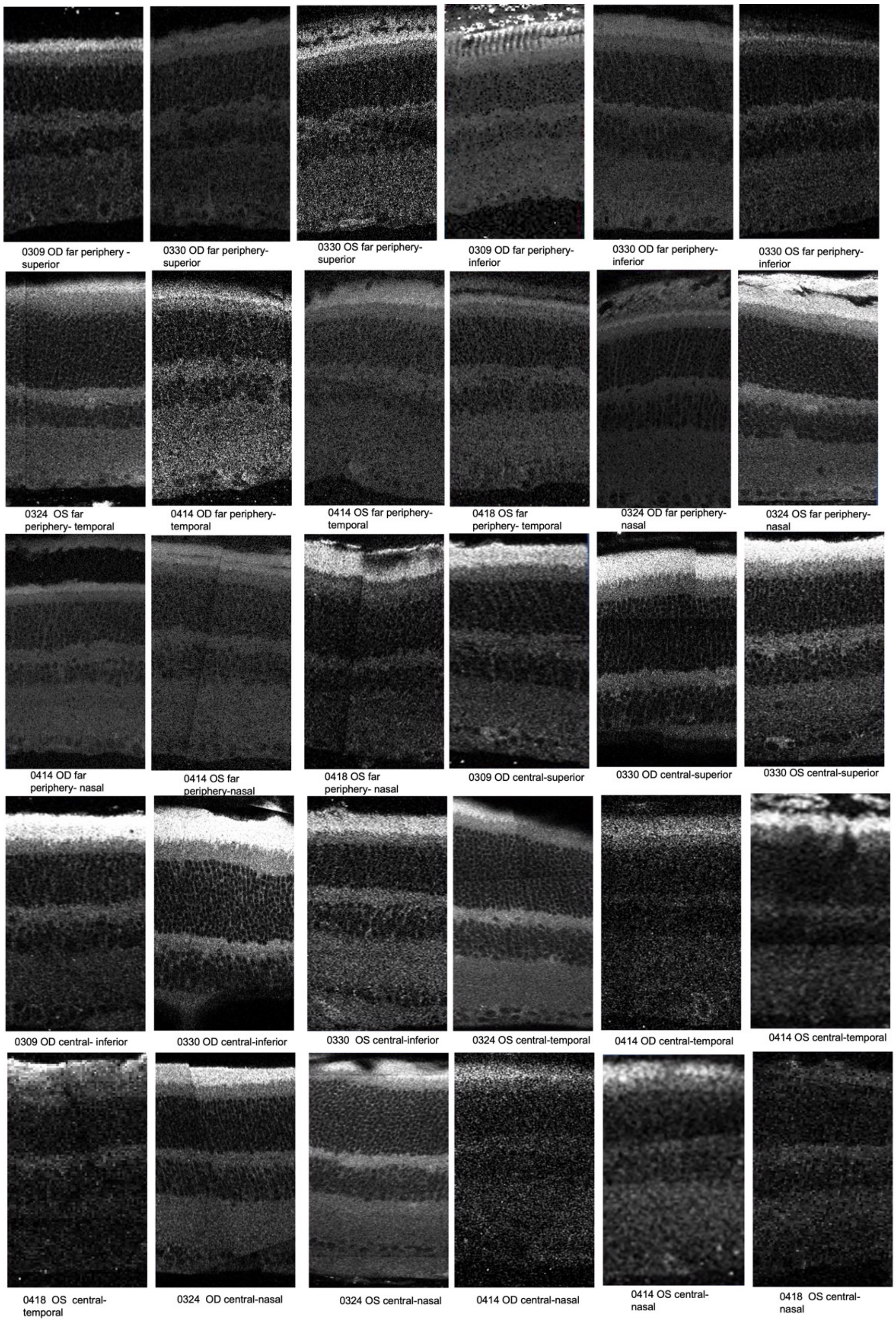

Figure S3. Intensity images of far periphery and central zones.

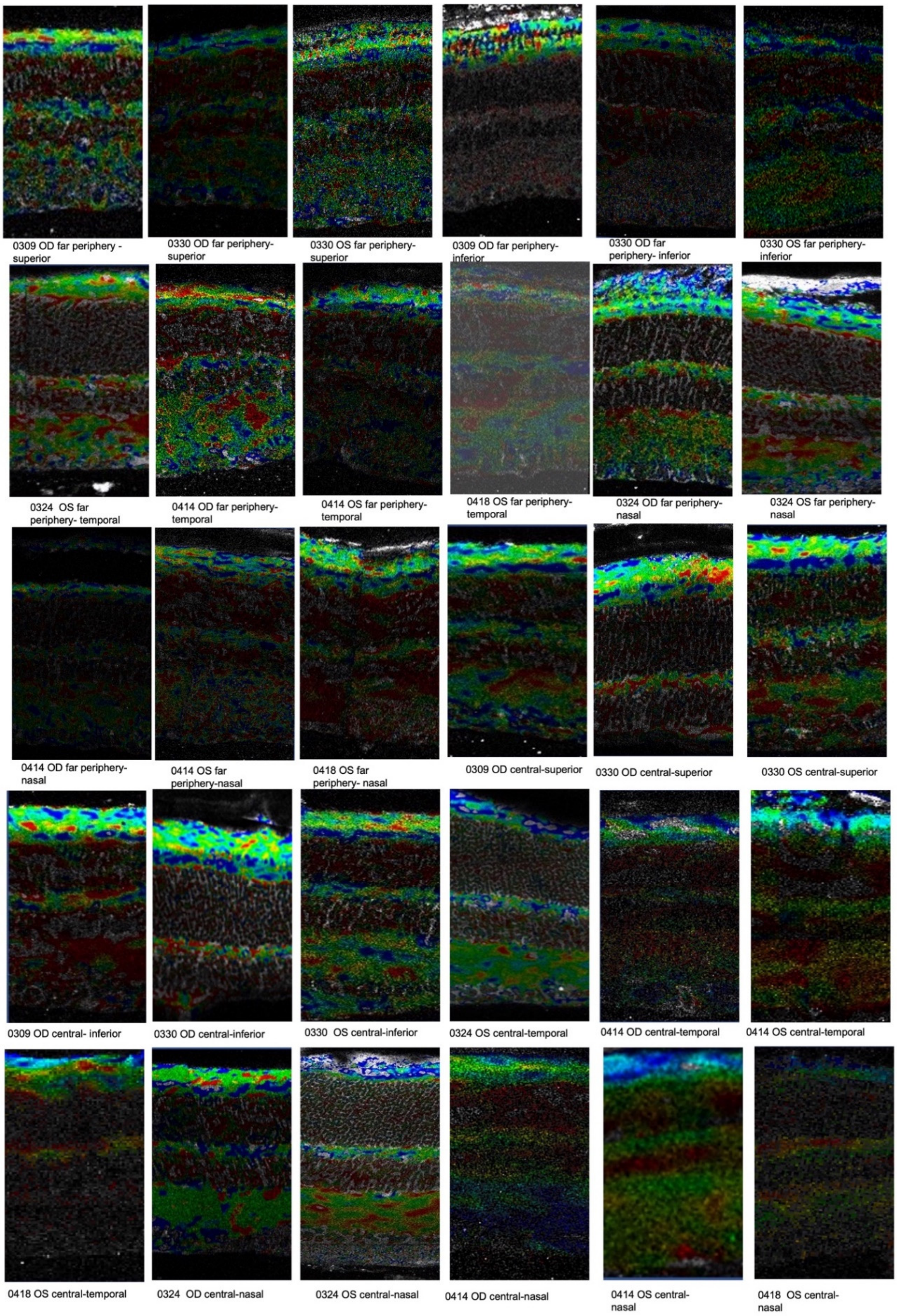

Figure S4. A2 distribution images of far periphery and central zones.
